# Analysis for Stroke Etiology in Duplicated/Accessory MCA-Related Cerebral Infarction: Two Case Report and Brief Literature Review

**DOI:** 10.3390/diagnostics11020205

**Published:** 2021-01-30

**Authors:** Kou Tsuyama, Nobukazu Miyamoto, Atsuhiko Shindo, Kenichiro Hira, Yuji Ueno, Kenji Yatomi, Hidenori Oishi, Nobutaka Hattori

**Affiliations:** 1 Department of Neurology, Juntendo University Hospital, 3-1-3 Hongo, Bunkyo, Tokyo 113-8431, Japan; k-tsuyama@juntendo.ac.jp (K.T.); at-shindo@juntendo.ac.jp (A.S.); knhira@juntendo.ac.jp (K.H.); yuji-u@juntendo.ac.jp (Y.U.); nhattori@juntendo.ac.jp (N.H.); 2 Department of Neurosurgery, Juntendo University Hospital, 3-1-3 Hongo, Bunkyo, Tokyo 113-8431, Japan; kyatomi@juntendo.ac.jp (K.Y.); ohishi@juntendo.ac.jp (H.O.)

**Keywords:** duplicated/accessory middle cerebral artery, cerebral infarction, dissection, embolism, magnetic resonance angiography, digital subtraction angiography

## Abstract

Duplication and accessory of the middle cerebral artery (MCA) constitute a rare congenital variation. MCA anomalies are found at a lesser frequency than the vascular anomalies of the other major intracranial arteries. Duplicated/accessory MCA was usually noted incidentally with subarachnoid hemorrhage, due to resulted aneurysmal formation. However, duplicated/accessory MCA-related cerebral infarction is rarer. We report two cases of cerebral infarction due to dissection at the entry of the duplicate/accessory MCA. Both cases were similar in dissected site and clinical course, without headache or injury. In 20 previously reported cases and our two cases of duplicated/accessory MCA-related infarction, mean age (55.8 ± 21.2 years) was slightly younger for cerebral infarction, and stroke etiology was mainly embolism. The main etiologies of stroke were embolism and dissection. Considering embolism etiology, proximal site of arterial diameter changing lesion was a common site for embolism, as duplicated/accessory MCA was usually smaller than normal M1 segment. In cerebral dissection cases, the dissected site was similar to our cases. Numerous mechanisms of dissection were considered, but they mainly included dysfunction of the media and endothelium or shearing stress at the entry of duplication. As the detailed mechanisms of cerebral dissection remain unknown, clinicians should include a differential diagnosis for MCA dissection.

## 1. Introduction

The middle cerebral artery (MCA) is the most complex and largest cerebral artery because the human cerebral neocortex is significantly differentiated and developed. The MCA can be considered as a collateral branch of the anterior cerebral artery (ACA) and is a recent phylogenetic acquisition [1]. MCA anomalies occur less frequently than those of the other major intracranial arteries (ex. origin of the ACA, posterior cerebral artery). MCA anomalies in humans were reported in 0.2–4.0%, consisting of accessory MCA, duplicated MCA, duplicated origin of the MCA and fenestration of the MCA [1]. Crompton first defined the term “accessory MCA” in 1962, describing it as a vessel that passes along with the MCA into the Sylvian fissure [1]. After several decades, the term accessory MCA was redefined as a duplicated/accessory MCA. In 1973, Teal et al. proposed that the term accessory MCA was restricted to the anomalous artery that originates from the ACA and that the duplicated MCA is the branch originating from the ICA. Today, Teal’s classification is widely accepted. The incidence of accessory MCA is 0.3–4.0% and that of duplicated MCA is 0.2–2.9% by anatomical and angiographic analysis [2]. Komiyama et al. suggested that the development of an accessory and/or duplicated MCA is an anomalously early ramification of the early branches of the MCA because they have a consistent cortical supply through the accessory and duplicated MCA to the anterior frontal lobe and anterior temporal lobe, respectively [2].

Duplicated/accessory MCA-related subarachnoid hemorrhage, as the result of aneurysmal formation, was reported in 29 cases between 1970 and 2015 [3,4]. Direct surgery was performed in 18 ruptured cases and only one case was performed by intentional partial coil embolization. Additionally, in the unruptured case, direct surgery was carried out, except one case was treated with endovascular surgery. Most of the aneurysms were small (less than 10 mm), except 2 large cases (10 mm or more, less than 25 mm). There were no giant aneurysms (25 mm or more). Wide-necked aneurysms were found in 20 of 25 cases. In some cases, the aneurysm arises solely from the duplicated MCA rather than the junction of the internal carotid artery and the duplicated MCA [4,5,6].

In ischemic cases of relating with duplicated/accessory MCA, 12 cases were reviewed by Matsunaga et al. [7], and the etiology in this case series was three cardioembolic cases, four arteriosclerosis cases and four embolic cases (one unknown). Only Uchino et al. previously reported duplicated MCA infarction caused by dissection [8]. Therefore, duplicated/accessory MCA-related infarction with cerebral artery dissection is relatively rare. We report two cases of cerebral infarction related to duplicated/accessory MCA dissection without injury.

## 2. Case Report

### 2.1. Case 1

**Figure 1:**
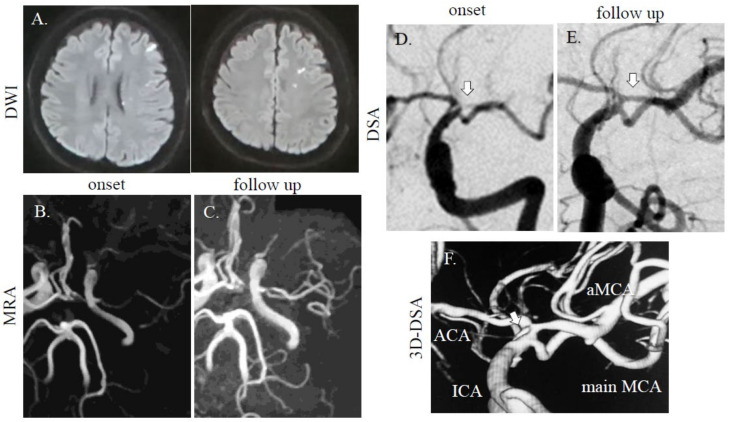
MRI/A and digital subtraction angiography (DSA) images of cases 1. (**A**) A dotted high-intensity area was observed in the left middle cerebral artery (MCA) distal area. In case 1, the left MCA was faint at onset (**B**), but accessory MCA was noted on follow-up (**C**) on MRA. On DSA, one intact MCA and one stenotic MCA (arrow) were found (**D**). In the follow-up study, stenotic MCAs were recovered (**E**). On 3D-DSA, dissection scar was still noted at origin of main/accessory MCA (**F**; arrow). Diffusion-weighted images (DWI); repetition time (TR), 7000 ms; echo time (TE), 120 ms. MRA (without using contrast agent); TR, 30 ms; TE, 6.8 ms.

A 40-year-old healthy Asian man with bronchial asthma and no vascular risk factors suddenly developed right hemiparesis and aphasia without headache. He had no history of collagen disorders. He was brought to our hospital 60 min after onset (National Institutes of Health Stroke Scale (NIHSS) score of 16 at arrival; motor aphasia, dysarthria, right facial paresis, and right flaccid hemiparesis), but blood tests were normal including blood coagulation system. Cerebral magnetic resonance imaging (MRI) revealed a dotted acute infarct area in the left hemisphere on diffusion-weighted images (DWI) and string signs were observed at the top of the internal carotid artery (ICA). Distal left MCA flow disappeared due to proximal MCA stenosis on magnetic resonance angiography (MRA) (Figure 1A,B), suggesting cerebral artery dissection. Thrombolytic therapy was prepared. As his symptoms improved after MRI (NIHSS 3; dysarthria, sensory disturbance, and ataxia of the upper limb), thrombolytic treatment was not performed, due to improvement symptoms. On digital subtraction angiography (DSA) for potential thrombectomy, we found a duplicated/accessory MCA. The superior segment was string-like and ended in the anterior cerebral artery (ACA) and the inferior segment continued as the main MCA stem with distal patent branches (Figure 1D); therefore, we were unable to perform thrombectomy. We selected dual antiplatelet therapy (aspirin at 200 mg and clopidogrel at 75 mg with loading on the first day (300 mg)), and intravenous drip of edaravone and hydration, which improved his symptoms. The follow-up MRA/DSA revealed persistent dissection of the left MCA, but with improved distal MCA flow (Figure 1C,E). However, on 3D-DSA, dissection scar was still noted at the origin of the main/accessory MCA (Figure 1F). At discharge, his symptoms were ameliorated. Three months later, he had no neurological deficit.

### 2.2. Case 2

**Figure 2:**
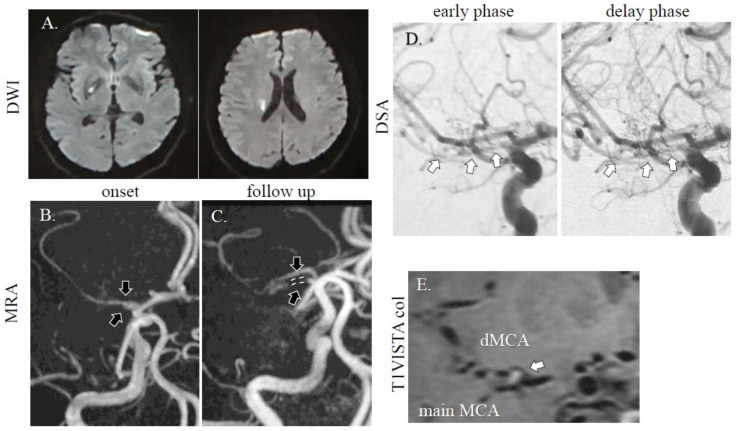
MRI/A and DSA images of case 2. A dotted high-intensity area was observed in the right basal ganglia area (**A**). The right MCA was faint (**B**; downwards arrow: intact MCA, upwards arrow: stenotic MCA), and there was one intact MCA and one stenotic MCA (**C**; downwards arrow: intact MCA, upwards arrow and dotted line: stenotic MCA). On DSA, a duplicated MCA was observed (**D**; arrows: stenotic MCA). On T1-VISTA colonal (col) image, intramural hematoma was found in the origin of duplicated MCA (**E**; arrow). DWI; TR, 7000 ms; TE, 120 ms. MRA (without using contrast agent); TR, 30; TE, 6.8 ms. T1-VISTA; field-of-view (FOV): 120 mm, TR: 400 ms, TE: 10ms, volume-of-interest (VOI) size: 1 × 1 × 1 mm, 100 slices.

A healthy 56-year-old Asian man without vascular risk factors suddenly developed left body dysesthesia and walking difficulty at midnight. He had no history of collagen disorders. On arrival to the emergency room (8 h after onset), his NIHSS score was 1 (sensory deficit on the left) without headache, and blood tests were unremarkable, including blood coagulation system. MRI/A revealed acute cerebral infarction at the right basal ganglia (Figure 2A,B), and an intact MCA and stenotic MCA (distal flow of the stenotic MCA was not detected). He received dual antiplatelet therapy (aspirin at 200 mg and clopidogrel at 75 mg with loading on first day (300 mg)) and intravenous drip of edaravone, which improved his symptoms. On DSA (for diagnosis, performed 1 week after stroke onset), duplicated/accessory MCA was noted with one stenotic MCA (Figure 2D). On follow up MRI performed after day 8, the ischemic area did not expand and visualization of the stenotic MCA on MRA can be observed (Figure 2C). On the T1-weighted volume isotropic turbo spin-echo (T1-VISTA) image, intramural hematoma was found in the origin of duplicated MCA (Figure 2E). At discharge, he had no neurological deficit. On outpatient clinic at 3 months after admission, he did not have new neurological deficit, but MCA visualization was not improved.

## 3. Discussion

Between January 2016 and December 2019, 813 patients with cerebrovascular diseases, including ischemic and hemorrhagic stroke, were admitted to the Department of Neurology, Juntendo University Hospital. We found only two patients present met the criteria for duplicated/accessory MCA from our hospital (0.24%). In addition, we reviewed published scientific reports by searching the PubMed database. The keywords used were “accessory MCA”, “duplicated MCA” and “cerebral infarction”. We reviewed related articles for all cases. We also excluded cases before December 1999, because MRA was not common and mechanical differences, but Komiyama et al.’s article [2] was included because angiographical analysis was clearly performed. The same variables were collected from the previously reported cases if available (ex. vascular risk factors, demographic data, initial treatment/final antiplatelet therapy and MRI/MRA findings). We found 20 cases from the literature search. The clinical presentation, radiological findings, and vascular risk factors for the 22 cases (including two cases from this study) are described in the Table 1.

The mean age was 55.8 ± 21.2 years (mean ± standard deviation; range, 21–84 years), was slightly younger for cerebral infarction, and most of the patients were male (15/22; 68.1%). Vascular anomaly type was mainly accessory type (17/22; 77.2%). The infarct sites were varied, but ischemic lesion was multiple and involved cortex. The clinical severity was relatively mild (NIHSS < 8: 8/12; 66.6%), and stroke prognosis was relatively good (mRS < 3: 14/18; 77.7%) in accessory/duplicated MCA-related stroke. The variation of initial treatment was mainly performed by hyperacute treatment (thrombolytic therapy [(4/17; 23.5%), interventional radiology; thrombectomy (5/17; 29.4%)].

Stroke etiology was mainly by embolism (9/19; 47.3%, cardiogenic embolism (6/19; 31.5%), embolic stroke undetermined source (ESUS 3/19; 15.7%)). In addition, next to high frequency was cerebral artery dissection and atherothrombotic infarction (4/19; 21.0%). Generally, common site of embolism is proximal site of changing arterial diameter, for example, top of ICA, bifurcation of MCA, top of basilar artery and arteriosclerotic lesion. As duplicated/accessory MCA, because the artery diameter was usually smaller than the normal M1 segment [1], therefore it may be suggested that embolism was high frequency compared to another etiology. At the duplicated/accessory MCA-related dissection development, many hypotheses have been proposed. Similar to cerebral artery bifurcations, the elastin discontinuity with thinned sub-endothelium may be important for aneurysm formation [23]. Based on neuropathological analysis, dissection is generally due to disruption of the internal elastic lamina and the media, similar to aneurysms [24]. In addition to the micro-histological factors, the hemodynamic effects, to which the arterial wall of the duplicated artery is directly exposed (such as shear stress from the blood pressure), play an important role in the development of dissection and aneurysms [25]. An embryological and morphological study is needed, and clinicians must consider MCA dissection as a differential diagnosis.

In 22 cases, the infarction of the perforating artery area was involved in duplicated/accessory MCA-related infarction 77.7% of the time (14/18). On the contrary, in angiographical analysis in the 1980s, Abanou et al. [26] and Lasjaunias et al. [27] found that the duplicated MCA is a pure cortical vessel without perforating arteries. However, in line with our analysis, Umansky et al. [28] reported that both the duplicated MCA and the main MCA have perforating arteries to the anterior perforated substance on microanatomical study. Crompton [29] observed that the duplicated MCAs occasionally have perforating arteries. Komiya et al. also noticed that the perforating artery arose from the duplicated MCA in three cases [2]. Uchino et al. hypothesized that the duplicated origin of the MCA is formed by distal fusion of the duplicated MCA or accessory MCA, and collateral anastomoses existed between the accessory/duplicated MCA and main MCA trunk through leptomeningeal anastomoses and perforating arteries [30]. Nowadays, it is generally accepted that the accessory MCA frequently has perforating arteries to the anterior perforated substance [1]. Although the extent of vascular supply may vary, as basal ganglia was included in ischemia in our cases, duplicated MCA may have perforated the basal ganglia indirectly.

The vascular variation of the MCA is a difficult problem in the treatment of stroke in the hyperacute phase. As thrombectomy is the only method to improve the symptoms of hyperacute cerebral infarction, this procedure may be performed more for such patients in the future [31]. Therefore, opportunities to treat patients having concomitant vascular variations may increase. A previous report described a patient who presented with discrepant clinical symptoms and imaging findings, and underwent mechanical thrombectomy, revealing a duplicated/accessory MCA [18]. In cases of vascular variation, endovascular therapy can lead to undesirable consequences [20]. Before endovascular intervention, detailed evaluation of hemodynamics should be considered, especially when the patient has both normal and infarcted regions by marked contrast in the same MCA area, regardless of occlusion of the ICA (unusual presentation of cerebral infarction). These patients may possess a duplicated/accessory MCA and the occlusion of one artery may have caused infarction in only part of the MCA [20]. Clinicians should consider MCA vascular variations.

We acknowledge that these types of studies have limitations regarding selection and positive outcome biases. However, we believe that the data obtained should be considered as they may lead to safe and efficient treatment methods for patients for whom an appropriate treatment has yet to be established. Second, we were unable to retrieve information about the duration of treatment or compliance with daily medication considering the retrospective nature of this study.

## 4. Conclusions

In conclusion, we reviewed duplicated/accessory MCA-related infarction. In our cases, MRI/A analysis was performed in detail and during follow-up. As the detailed mechanisms of cerebral dissection remain unknown, clinicians should include a differential diagnosis for MCA dissection.

## Figures and Tables

**Table 1:** Clinical features duplicated/accessory MCA-related cerebral infarction.

Author (et al.)	Age	Sex	Arrhythmia	Duplicated (D) /Accessory(A)	Vascular Risk Factors	Infarct Lesion or Symptom (on No Ischemic Lesion/TIA Case)	Perforator Area Involved	Stroke Etiology	Initial Treatment	NIHSS	Outcome (mRS)
Our Case 1	40	M	none	A	none	Frontal, corona-radiata	O	Dissec	Asa Clo	16 > 3	1
Our Case 2	56	M	none	D	none	basal ganglia	O	Dissec	Asa	1	0
Komiyama [2]	63	M	paf	A	ND	temporal, basal ganglia	O	CE	ND	ND	ND
	71	M	Af	A	ND	frontal, temporal, parietal	X	CE	ND	ND	ND
Uchino [8]	13	M	none	D	none	temporal	X	Dissec	ND	ND	0
Moriente [9]	53	M	ND	A	ND	TIA (sensory-motor)	ND	ND	medical	5 > 0	0
	21	F	ND	D	ND	basal ganglia	O	Dissec	ND	ND	0
Gao [10]	63	M	none	A	ND	Aphasia + right hemiparesis (no ischemic lesion)	X	ATBI	DAPT	ND	0
Menon [11]	58	M	ND	A	ND	corona-radiata, temporal	O	ESUS	medical	14	ND
Kuwahara [12]	27	M	ND	A	ND	hemiparesis (no ischemic lesion)	ND	ND	IV-tPA	ND	0
Oshikata [13]	59	M	none	A	HT DL DM	corona-radiata	O	ATBI	heparin DAPT Ed	3 > 8	3
Gómez-Choco [14]	53	F	ND	D	ND	TIA (left hemiparesis)	ND	ND	ND	ND	0
Hiramatsu [15]	60	M	none	A	DM DL smoke	temporal, corona-radiata	O	ATBI	Heparine > Asa	3 > 0	0
	81	F	none	A	HT DL	frontal, corona-radiata	O	ESUS	IV-tPA > VKA	8 > 4	2
Nomura [16]	64	M	none	A	DM HT	corona-radiata	O	ATBI	medical	ND	0
Noguchi [17]	64	M	none	A	HT DM	TIA (aphasia + dysarthria)	ND	ATBI	STA-MCA bypass	0	0
Bayer-Karpinska [18]	21	F	ND	A	none	basal ganglia, corona-radiata	O	ESUS	IV-tPA IVR	ND	ND
Liu [19]	77	F	Af	A	ND	temporal, basal ganglia	O	CE	IV-tPA	12 > 2 > 0	0
Matsunaga [7]	82	M	Af	A	ND	temporal, parietal, basal ganglia	O	CE	IVR	17	3
Deguchi [20]	84	M	Af	A	HT DM	temporal, parietal, basal ganglia	O	CE	IVR	15	6
Cookie [21]	37	F	none	A	ASD	basal ganglia	O	CE	IVR	ND	0
Koge [22]	82	F	none	D	none	temporal, parietal, basal ganglia	X	ESUS	IVR	28	6

Af, atrial fibrillation; Asa, aspirin; ASD, atrial septal defect; ATBI, atherothrombotic brain infarction; CE, cardiogenic embolism; Clo, clopidogrel; Cil, cilostazol; Dissec, dissection; DL, dyslipidemia; DM, diabetes mellitus; Ed, edaravone; ESUS, embolic stroke undetermined source; HT, hypertension; ND, not described; NIHSS, National Institutes of Health Stroke Scale; mRS, modified Rankin Scale; TIA, transient ischemic attack; IV-tPA, intravenous tissue plasminogen activator; IVR, interventional radiology; Paf, paroxysmal atrial fibrillation; VKA, vitamin K antagonist; O, involved; X, not involved.

## Data Availability

The data that support the findings of this study are available from the corresponding author upon reasonable request.
